# Nutrition Concepts for the Treatment of Obesity in Adults

**DOI:** 10.3390/nu14010169

**Published:** 2021-12-30

**Authors:** Meike Wiechert, Christina Holzapfel

**Affiliations:** Institute for Nutritional Medicine, School of Medicine, Technical University of Munich, 80992 Munich, Germany; meike.wiechert@tum.de

**Keywords:** dietary recommendation, weight loss, overweight, intermittent fasting, carbohydrate, fat, protein

## Abstract

Obesity caused by a positive energy balance is a serious health burden. Studies have shown that obesity is the major risk factor for many diseases like type 2 diabetes mellitus, coronary heart diseases, or various types of cancer. Therefore, the prevention and treatment of increased body weight are key. Different evidence-based treatment approaches considering weight history, body mass index (BMI) category, and co-morbidities are available: lifestyle intervention, formula diet, drugs, and bariatric surgery. For all treatment approaches, behaviour change techniques, reduction in energy intake, and increasing energy expenditure are required. Self-monitoring of diet and physical activity provides an effective behaviour change technique for weight management. Digital tools increase engagement rates for self-monitoring and have the potential to improve weight management. The objective of this narrative review is to summarize current available treatment approaches for obesity, to provide a selective overview of nutrition trends, and to give a scientific viewpoint for various nutrition concepts for weight loss.

## 1. The Challenge Obesity

Obesity is a complex, multifactorial, and largely preventable chronic disease defined as abnormal or excessive fat accumulation [1]. Body mass index (BMI), calculated as weight in kilograms divided by the square of height in metres (kg/m^2^), is the current most widely used criterion for classifying obesity [1]. People with a BMI ≥ 25 kg/m^2^ are classified as overweight, and a BMI ≥ 30 kg/m^2^ is categorized as obese [1,2,3] (Table 1). Although the BMI captures the degree of overweight and obesity, abdominal obesity measured mainly by waist circumference is additionally associated with health risks (Table 1). Clinical practice and medical guidelines focus on BMI and waist circumference as simple, objective, and reproducible tools to measure weight status and abdominal obesity. However, the diagnosis of obesity should not be based on BMI alone, rather together with other anthropometric and clinical parameters. Instrumental methods (e.g., bioimpedance analysis, Dual Energy X-ray Absorptiometry, and magnetic resonance imaging) to assess body composition and adipose tissue depots are available but are often time- and cost-intensive.

It is well known that overweight and obesity are the main risk factors for several diseases such as type 2 diabetes mellitus, hypertension, dyslipidaemia, cardiovascular disease, and several types of cancer [4]. Recently published data show that BMI is positively associated with severe coronavirus disease 2019 (COVID-19) outcomes [5]. Furthermore, an increased BMI might lead to a decline in quality of life and contribute to a decreased life expectancy [6,7,8,9].

The cause of obesity is a long-term energy imbalance caused by a combination of increased energy intake and reduced energy expenditure [1,6,10,11]. The National Health and Nutrition Examination Survey (NHANES) observed that the average daily energy intake increased from 1971 to 2000 in men by 168 kilocalories (kcal)/day and women by 335 kcal/day [12]. Without an active regulation or adaptation of energy balance, this increase theoretically could explain a weight gain per year of eight kilograms for men and 16 kg for women. Furthermore, energy expenditure has decreased over the last decades [12]. Basset and colleagues reported that in 2003, an American adult walked about 5000 steps/day. Compared to people living 300 years ago, it is a difference of 13,000 steps/day for men and 9000 steps/day for women [13]. Without any physiological adaption, this decline in physical activity could explain a yearly weight gain of 31 kg for men and 21 kg for women [10]. Daily occupation-related energy expenditure has decreased by more than 100 kcal/day in U.S. adults. That alone could explain a substantial weight gain in the population over the last five decades [14]. In addition to lifestyle factors, other contributing factors like food and environment have been identified [15].

Over the past 50 years, the prevalence of obesity has increased worldwide in pandemic dimensions [3,6,16,17,18]. The Global Burden of Disease study with data from 68.5 million persons demonstrated that in 2015 603.7 million adults were obese [19]. Since 1980, the prevalence of obesity has doubled in more than 70 countries and has continuously increased in most other countries [19]. If trends continue, by 2030, an estimated 38% of the world’s adult population will be overweight, and another 20% will be obese [20]. The confinements by the COVID-19 pandemic have changed lifestyle behaviour and have promoted an obesogenic environment. Weight trajectories during the COVID-19 lockdown have been shown [21,22]. It is expected that the COVID-19 pandemic reinforces the obesity pandemic with long-term consequences on the prevalence of overweight and obesity [23]. Because of the rapid increase in the prevalence and disease burden of obesity, it is indispensable to focus on monitoring BMI and to identify, implement, and evaluate evidence-based interventions to address this health issue [19].

## 2. Treatment Approaches

**Figure 1:**
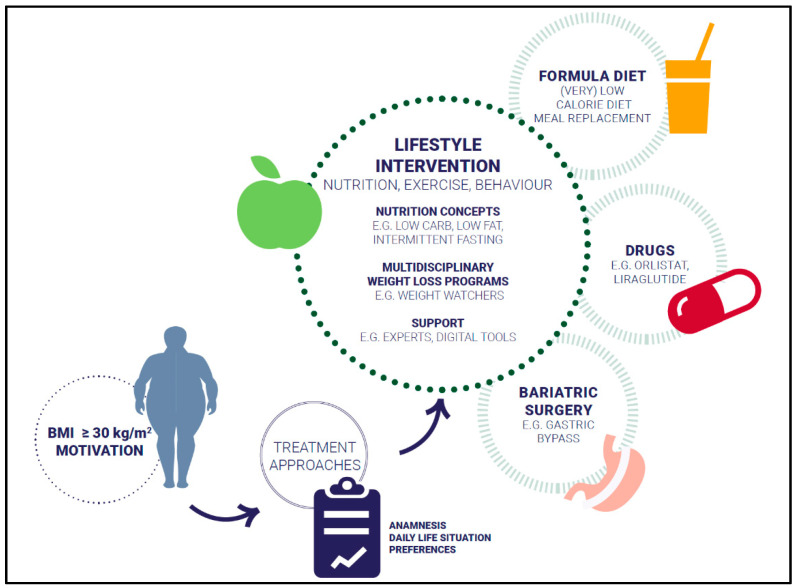
Treatment approaches.

**Figure 2:**
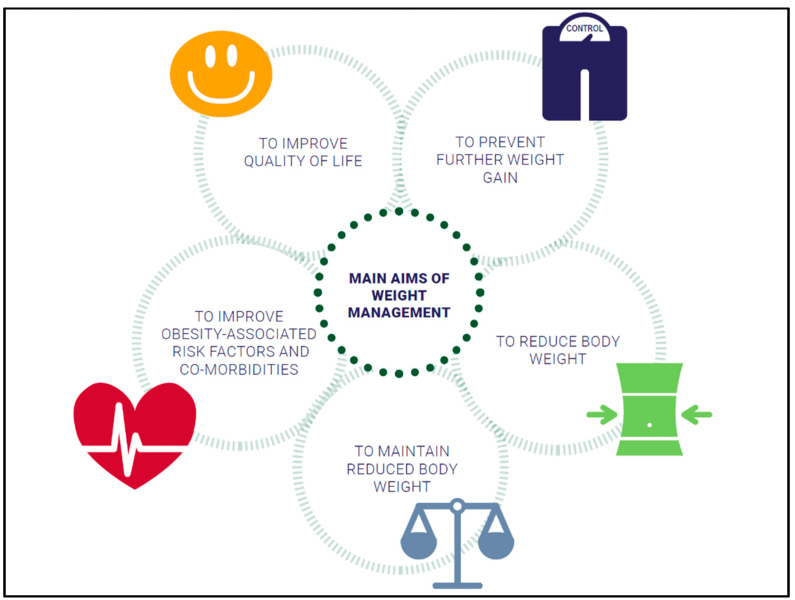
Overview of weight management goals.

Different evidence-based approaches considering weight history, BMI category, and co-morbidities to treat overweight and obesity are available: lifestyle intervention, formula diet, drugs, and bariatric surgery (Figure 1). The main aims of weight management are shown in Figure 2.

### 2.1. Lifestyle Intervention

Lifestyle interventions inducing a negative energy balance provide the basis for the treatment of overweight and obesity and are part of the standard recommendation. Different lifestyle approaches exist, whereas nutrition, physical activity, and behaviour are the main components. By lowering energy intake and increasing physical activity accompanied by behavioural change techniques, a daily energy deficit of about 500 kcal is recommended for weight loss. This energy deficit can produce a moderate weight loss over one year. Energy balance changes with weight loss, making it necessary to adjust energy intake and expenditure during weight management. Energy balance is dynamic and weight loss leads to a new energy equilibrium on a lower level. Adherence to a lifestyle intervention is challenging for many people with overweight and obesity. In a systematic review and meta-analysis, three main factors were associated with improved adherence to weight loss interventions: supervision, social support, and focus on dietary intervention [24].

Nutrition is the main lifestyle factor. Therefore, nutrition aspects to decrease energy intake and to support weight management are highlighted in the following.

#### 2.1.1. Energy Intake

Key components of energy balance include energy intake, expenditure, and storage. When energy intake exceeds energy expenditure, a state of positive energy balance increases body weight. The European Food Safety Authority (EFSA) recommends a daily dietary reference intake of 45% to 65% of total kilocalories from carbohydrates, 20% to 35% from fat [25], and 0.83 g protein/kg body weight [26]. For weight loss, a daily energy deficit of 500 kcal is recommended and can be reached by avoiding energy-dense food. Fat is a high-energy macronutrient and provides more than twice as high as the energy of carbohydrates or protein. Because of this, a reduction in daily fat intake supports to lower daily caloric intake. Fat intake can be reduced by using low fat dairy products like cheese, and yogurt, lean meat, and avoidance of hidden fats. In Table 2, recommendations and practical examples are shown for decreasing energy intake.

#### 2.1.2. Macronutrients

Studies have shown that not the macronutrient composition of the diet but the energy content is relevant for weight management [27,28].

Low carb diets often contain approximately 40% of carbohydrates per day. The lowest intake of carbohydrates is part of a ketogenic diet, where the aim is to minimize the carbohydrate intake as much as possible. Epidemiological data showed that a daily amount of carbohydrates of 50 to 55% correlates with the lowest mortality rate. Low carb diets, as well as high carb diets, increase the mortality risk (U-shaped association) [29]. A low carb diet includes a lower amount of plant-based food that has health-promoting effects. A meta-analysis with eight randomized controlled studies concluded that low carb diets are superior to diets with a low amount of fat regarding lipid metabolism in people with overweight and obesity [30]. However, data from NHANES indicate that realized decreases in the percentage of energy consumed from fat were associated with increased total energy intake caused by compensatory over-consumption of energy from sugars [31]. The stone age diet or paleo diet is a low carb diet, but also not clearly defined. In general, it is a diet with a high amount of meat and protein. The variety of foods is limited by the renouncing of grain. Smaller short-term intervention studies with methodological limitations examined the effects of a paleo-conform diet. Manheimer et al. evaluated data of four randomized controlled trials (RCTs) with 159 participants that compared the palaeolithic diet with any other dietary pattern. Results showed that palaeolithic nutrition resulted in stronger short-term improvements of cardiovascular risk factors like waist circumference and blood pressure than control diets [32]. In 70 post-menopausal women with obesity, it has been shown that there was no significant difference between a palaeolithic-type diet and the Nordic nutrition recommendations in anthropometric changes after 24 months [33].

An evaluation of almost 50 studies found that the participants, regardless of the macronutrient composition of the diet, lost the same amount of weight within 6 and 12 months of intervention [34]. In another study, 811 persons were randomized into four groups of diets with a different energy intake from fat, protein, and carbohydrates (20, 15, and 65%; 20, 25, and 55%; 40, 15, and 45%; 40, 25, and 35%, respectively). After two years of intervention, the weight loss was about 4 kg (completers-analysis), with no significant differences between the groups [28]. Furthermore, the comparison of three different diets (low fat/low energy diet; Mediterranean/low energy diet; low carb/non-energy reduced diet) resulted in similar findings. Mean weight loss after 2 years of intervention was 3.3, 4.6, and 5.5 kg, respectively (completers-analysis) [27]. In a study with 609 adults with BMI between 28 and 40 kg/m^2^, the mean weight loss was about 6.0 kg (low carb diet) and 5.3 kg (low fat diet) after one year of intervention [35]. A systematic review of systematic reviews comparing low carb diets with control diets on weight loss concerned the low quality of studies, and concluded that better quality reviews and RCTs are needed for a clear recommendation of low carb diets as preferred to other energy-reduced diets [36]. Even with the plant-based form of Atkins diet or the Mediterranean diet, there is a moderate weight loss [27,37,38]. A meta-analysis indicates that a Mediterranean diet low in energy leads to moderate weight loss [39].

Finally, the macronutrient composition of a diet has no major impact on weight loss. Low carb, as well as low fat concepts, are effective for weight loss if a negative energy balance is provided.

A meal replacement is a high protein product used to replace at least one main meal per day. Those products are permitted to be marketed for weight management purposes and have specific regulatory requirements concerning supplementation with vitamins, minerals, and trace elements, as well as energy content, per portion. They are available e.g., as shakes, soups, or meal bars. Meal replacement strategy followed by a dietary change and behaviour modification strategy is popular among people trying to lose weight. One option is a very low calorie diet (VLCD) with < 800 kcal/day by total meal replacement. The other option is a low calorie diet (LCD) supplying > 800 kcal/day, generally in the range of 1200–1600 kcal/day. In a systematic review and meta-analysis on VLCDs, total weight loss ranged from 8.9 to 15 kg in persons with type 2 diabetes mellitus and 7.9 to 21 kg in persons without diabetes, over a treatment duration of 4 to 52 weeks. Study duration did not appear to influence overall weight loss. The average weight loss per week was about 0.5 kg [40]. Another review investigated the effect of weight loss interventions incorporating meal replacement compared with alternative interventions on weight change at 1 year in adults with overweight or obesity. In this review, studies with diets providing < 800 kcal/day, and with total diet replacement, were excluded. In general, all diets incorporating meal replacement resulted in a higher mean weight change at 1 year compared to the control groups or alternative diets [41]. The Diabetes Remission Clinical Trial (DiRECT) of 306 patients with type 2 diabetes mellitus demonstrated that diet-induced weight loss by total diet replacement (825–853 kcal/day formula diet for 3–5 months), followed by food reintroduction (2–8 weeks), and followed by structured support for long-term weight loss maintenance effectively reversed type 2 diabetes mellitus. At 12 months, 86% of the participants who achieved a weight loss >15 kg (24%) became drug-free and had remission of type 2 diabetes mellitus. An overall remission of type 2 diabetes mellitus in the intervention group was observed in 46% of patients after 1 year and in 36% of patients after 2 years [42,43].

#### 2.1.3. Intermittent Fasting

There are several approaches of intermittent fasting. The 16:8 concept is a form of time-restricted eating where individuals eat within a time window of 8 h and fast for the remaining 16 h daily. The 5:2 concept consists of a normal eating routine on 5 days per week, without any specific recommendations or restrictions, and 2 days of fasting with an energy intake of about 500 kcal. Conley M. et al. compared the 5:2 diet (2 non-consecutive days with 600 kcal and 5 days of energy intake ad libitum per week) with an energy-reduced diet. After 6 months of intervention, both groups reduced their body weight similarly (5.3 kg (5:2) vs. 5.5 kg (standard)) with no significant difference [44]. A RCT compared the effects of alternate-day fasting with daily caloric restriction on body weight in participants with obesity. Findings demonstrate comparable weight loss after 6 (alternate day fasting: 6.8%, caloric restriction: 6.8%) and 12 months (6.0% versus 5.3%) [45]. In a systematic review and meta-analysis of RCTs intermittent versus continuous energy restriction on weight loss and cardio-metabolic outcomes have been investigated. The included eleven trials with a duration from 8 to 24 weeks resulted in a similar weight loss between the two intervention groups [46]. Compared to a continuous energy restriction, intermittent fasting leads to similar weight loss and similar improvement of cardio-metabolic parameters [47,48,49]. The recently published Cochrane review by Allaf et al. found that people lost more weight with intermittent fasting concepts than without a special dietary concept over three months (evidence from seven studies in 224 people). If intermittent fasting concepts were compared with energy-restricted diets for 3 months (10 studies; 719 people) or longer (3 to 12 months; 4 studies; 279 people), this difference in weight loss is lost [50]. The energy restriction causes the positive effect of intermittent fasting on weight loss, not fasting as a stand-alone intervention [51,52].

Besides weight loss, fasting-specific effects on metabolic regulation or cardiovascular health are discussed. In lean persons, there is no statistically significant difference between daily energy restriction and alternate-day fasting with or without energy restriction concerning postprandial indices of cardio-metabolic health, gut hormones, or the gene expression in subcutaneous adipose tissue [51].

A small study with eleven participants with overweight, early time-restricted fasting (eating between 8 a.m. and 2 p.m.) was investigated for acute effects on glucose metabolism and gene expression. In comparison to the control group (eating between 8 a.m. to 8 p.m.) 24 h glucose levels and glycaemic excursions decreased, and ketones, cholesterol, and the expression of the stress response and aging gene sirtuin 1 (SIRT1) and the autophagy gene microtube associated protein 1 light chain 3 alpha (LC3A) increased in the morning before breakfast. This was different to the gene expression pattern in the evening [53]. The early time-restricted feeding effect on cardio-metabolic health (insulin sensitivity, beta-cell responsiveness, blood pressure, oxidative stress, and appetite)—independent from weight loss—has further been observed by Sutton et al. in men with prediabetes [54]. In addition, a RCT with 17 participants with normal weight compared the metabolic effects of breakfast and dinner skipping. Compared to the three meals per day control group, skipping breakfast or dinner increased energy expenditure. Furthermore, fat oxidation increased on the breakfast skipping day [55]. In a prospective cohort study, it has been shown that breakfast skipping is associated with a 21% increased risk for the development of diabetes mellitus type 2 [56].

#### 2.1.4. Personalized Nutrition

In the last years, concepts of personalized nutrition have been more focused, especially by commercial providers offering direct-to-consumer genetic testing. One of the main drivers for personalized dietary recommendations is the inter-individual variability of metabolic response on standardized meal challenges suggesting that personalized diets might successfully modify elevated postprandial blood glucose and its metabolic consequences [57]. In the Personalised Responses to Dietary Composition Trial (PREDICT1) with more than 1000 twins and unrelated healthy adults in the UK, large inter-individual variability in postprandial responses of blood triglycerides (103%), glucose (68%), and insulin (59%) following identical meals was observed. Various intrinsic and extrinsic factors could be identified as predictors of the inter-individual variability. In the following, scientific evidence of gene-based and microbiome-based dietary recommendations is summarized.

##### Gene-Based Dietary Recommendations

Gene-based dietary recommendations are based on individuals’ genetic make-up. The fact that body weight is, inter alia, genetically determined, and more than hundreds of genetic loci are identified for a relationship with anthropometric parameters [58], is underlining the assumption that even the inter-individual varying degree of success in weight loss indicates a genetic component [59]. The *fat mass and obesity-associated* (*FTO*) gene is the gene with the largest effect on body weight. The function of the *FTO* gene is not yet fully understood, whereas it is shown that the *FTO* gene inhibits brown adipose tissue genesis [60]. Numerous companies offer genotyping and provide recommendations for a healthy diet or weight loss. Furthermore, commercial offerings for deoxyribonucleic acid (DNA) methylation profiling started to emerge. These commercially available direct-to-consumer tests are in contrast to the lacking scientific evidence that genotypes are associated with weight loss.

In a recently published pooled analysis of weight loss data, it has been shown that single nucleotide polymorphisms have a minor role in the inter-individual variation of weight loss [61]. The Food4Me study has shown that including genotype information for dietary recommendations had no beneficial effect on weight loss [62]. The American Society of Dietetics and Nutrition clearly states that ”No significant differences in weight, body mass index (BMI; calculated as kg/m^2^), or waist circumference were observed when results of genetic testing were incorporated into nutrition counselling compared with counselling or care that did not incorporate genetic results” [63], and the “use of nutrigenetic testing to provide dietary advice is not ready for routine dietetics practice” [64]. Present research cannot provide adequate evidence that individuals with a defined genetic make-up benefit especially from specific dietary recommendations concerning weight loss [65]. A systematic review on gene–diet interactions on weight change concluded that there is no evidence that gene–diet interactions are a main determinant for obesity treatment success [66]. For that reason, more future human studies are required to prove the clinical evidence of gene-based dietary recommendations on weight loss [59]. Furthermore, the investigation of single nucleotide polymorphisms will be replaced by the investigation of polygenetic scores to characterize humans according to their genetic predisposition. Khera AV et al. have developed and validated a genome-wide polygenetic score for five diseases (coronary heart disease, atrial fibrillation, diabetes mellitus type 2, inflammatory bowel disease, and breast cancer) [67]. In a further data analysis, a polygenetic predictor for body weight has been developed and validated [68].

##### Microbiome-Based Dietary Recommendations

Various correlations between the gut microbiota and individuals’ nutrition, as well as the occurrence of diseases like obesity, have been shown [59]. These correlations indicate that personalized nutrition based on the microbiome is a further starting point for weight loss [59]. Increasing evidence suggests that changes in individuals’ microbiome during dietary intervention are person-specific, and this heterogeneity, in addition to individuals’ physiology, is due to a unique microbiome signature [69]. The integration of microbiome information in combination with other person-specific factors seems to have the potential for the understanding of complex interactions [70]. Von Schwarzenberg et al. demonstrated that a VLCD led to a decrease in bacterial abundance and restructuring of the gut microbiome. After this VLCD, the microbiota were transplanted to mice, which led to a decrease in body weight. This study reveals that diet–microbiome interactions modulate energy metabolism [71]. Independent from weight loss, there is no doubt that dietary intake influences gut microbiota structure [72]. In a literature review, the direct and indirect mechanisms behind the influence of the gut microbiome have been discussed. The composition of the microbiota, the presence of specific microbes, and their metabolic activity have to be considered in future human intervention studies to investigate the potential for targeting the microbiota for improving health [73]. Similar to the genetic direct-to-consumer tests, some companies offer the analysis of the microbiome for personalized dietary recommendations, whereas scientific evidence is still not given. The current knowledge about the microbiome’s role in diet-mediated effects on health is too limited to provide evidence for microbiome-based dietary recommendations [74].

#### 2.1.5. Weight Loss Programs

Multidisciplinary weight loss programs addressing nutrition, physical activity, and behaviour are available. Most of the programs are delivered in a single country or are regionally rolled out, e.g., by health insurances, health care providers, or companies. The largest global weight loss program is WW (formerly Weight Watchers). An international study has shown that the WW program resulted in a moderate weight loss at 12 and 24 months. Mean weight change at 12 months assessment was 6.65 kg (completers-analysis) [75] and mean weight loss after 2 years was 4.76 kg [76]. Furthermore, Johnston et al. have shown that persons using all provided intervention tools (weekly meetings, WW mobile application, and WW online tools) lose more weight than persons who selected not all three ways to access the treatment [77]. Another well-known program is OPTIFAST, which also provides scientific evidence for efficacy after 12 months. The 12 months OPTIFAST concept includes a total dietary replacement for three months followed by lifestyle recommendations and professional group sessions for further nine months. Comparing the effectiveness of the OPTIFAST program with a food-based dietary plan resulted in 10.5% versus 5.5% weight loss at 52 weeks [78]. In this study, there was an active weight maintenance phase, where meal replacement was allowed.

#### 2.1.6. Support

Self-monitoring of diet and physical activity provides an effective behaviour change technique for weight management [79] and is a core component of behavioural obesity treatment [80]. It has been demonstrated that dietary self-monitoring itself and the frequency of self-monitoring is linked to weight loss [81]. Furthermore, self-monitoring tends to positively impact weight loss when combined with other self-regulation techniques, such as goal setting and feedback [82,83,84]. Engagement rates for self-monitoring diet were higher in digital than in paper-based self-monitoring [81,85].

Digital tools like online tools and applications (apps), tracking technologies, or even internet-based support have become attractive for teaching and supporting long-term behaviour change techniques [86]. Carter et al. examined in a RCT the acceptability and feasibility of a self-monitoring weight management intervention provided by a smartphone app compared to a website and paper diary. Results showed a mean weight change at 6 months of 4.6 kg in the smartphone app group, 2.9 kg in the paper diary group, and 1.3 kg in the website group and additionally, the app was highly appreciated in satisfaction and acceptability [85]. Weight management apps may have positive effects on weight-related outcomes; although, the methodological quality of many studies is low [87,88]. A meta-analysis by Villinger et al. with more than 6300 participants showed that app-based mobile interventions can be effective for changing nutrition behaviours and nutrition-related health outcomes [89]. Digital tools like apps are a time- and cost-effective method for the collection of health-related data with the potential of wide distribution and scalability [85,90,91]. Although many apps are available for weight loss, digital offerings for weight management rarely include evidence-based strategies for behaviour change [92,93].

## 3. Drugs

There are several agents for the pharmacologic therapy of obesity leading to decreased appetite, gastric emptying, nutrient absorption, or increased satiety. Some of these have gained marketing authorization during the last six years, and some others are still under development process [94,95]. Currently, the European Medicines Agency (EMA) authorized three drugs (orlistat, naltrexone/bupropion, and liraglutide), and the US Food and Drug Administration (FDA) has approved four drugs (orlistat, phentermine/topiramate, naltrexone/bupropion, and liraglutide) for obesity treatment [96]. The purpose of using pharmacotherapy to manage obesity is to increase patient adherence to lifestyle changes and to overcome the biological adaptations that occur with weight loss [97].

Increasing evidence has shown that behaviour-based interventions with one anti-obesity medication can result in greater weight loss than usual care conditions only [96]. The efficacy of available anti-obesity drugs is often limited to a reduction of 5–10% of body weight over a 1-year period and weight loss typically does not occur for more than 6–8 months [98]. In a systematic review and network meta-analysis, five weight loss medications (orlistat, lorcaserin, naltrexone-bupropion, phentermine-topiramate, or liraglutide) were compared regarding efficacy on weight loss. In total, 28 RCTs with 29,018 patients were included. Study participants in the placebo group had a statistically significant lower odds ratio for achieving 5% weight loss after one year than participants taking drugs. Excess weight loss was 8.8 kg for phentermine-topiramate, 5.3 kg for liraglutide, 5.0 kg for naltrexone-bupropion, 3.2 kg for lorcaserin, and 2.6 kg for orlistat [99].

## 4. Bariatric Surgery

Bariatric surgery is appropriate for persons with severe obesity. Indications of bariatric surgery vary across countries. In most countries, the access to bariatric surgery is, e.g., restricted to persons for whom other weight loss measures have failed. There are two primary mechanisms: restriction and malabsorption of ingested food. A restrictive approach is physically limiting the quantity of food that can be ingested by limiting the size and capacity of the stomach while leaving the remainder of the gastrointestinal tract intact. Malabsorption of calories and nutrients occurs when a portion is bypassed or removed. Kilocalories and nutrients are less able to be absorbed because ingested food remains in the gut to a shorter distance. The International Federation for Surgery of Obesity and Metabolic Disorders (IFSO) has published an annual report of all bariatric surgery provided to the Global Registry [100]. Data from 51 countries with documented surgery between 2014 and 2018 were evaluated. The surgery procedure carried out most frequently was sleeve gastrectomy (46.0%) followed by Roux en Y gastric bypass (38.2%), one anastomosis gastric bypass (7.6%), and gastric banding (5.0%) [100]. Within the systematic review by Puzziferry et al., 29 clinical studies with 7971 patients were evaluated. The main finding was that gastric bypass resulted in greater weight loss than the gastric band [101]. A further systematic review and meta-analysis showed that all current bariatric procedures are associated with significant weight loss, but more long-term data are needed for one-anastomosis gastric bypass and sleeve gastrectomy [102]. Particularly worthy to mention is that patients with higher adherence rates to visits and behaviour changes before surgery are more likely to lose more weight after surgery [103]. In any case, good preparation before and after surgery is indispensable to ensure the best outcomes [104]. Several steps during the preoperative evaluation are necessary. These include the individual’s psychological fitness to undergo bariatric surgery, the professional nutritional assessment, and patient education to guide the patient towards dietary modifications that are necessary after surgery [104]. Nutritional deficiencies are a long-term clinical issue in patients through modifications to the gastrointestinal tract [105]. The Clinical Practice Guidelines of the European Association for Endoscopic Surgery (EAES) recommend postoperative nutritional and behavioural advice for patients undergoing bariatric surgery [106]. Nutritional monitoring is an essential component of weight management after bariatric surgery to increase the patients’ adherence to healthy dietary habits and to appropriate supplementation measures [105]. In addition, monitoring prevents the risk of weight regain, makes it easier to detect possible nutritional deficiencies, and contributes to the preservation of a good quality of life [105].

## 5. Conclusions

This scientific viewpoint is a narrative review and not comparable with a systematic review but gives an overview of various treatment approaches, which should be used and combined considering the individuals‘ needs, preferences, weight status, and cardio-metabolic risk factors. All treatment approaches have to result in a negative energy balance. Independent of the weight loss concept (e.g., intermittent fasting, low carb, low fat, drugs or, bariatric surgery), weight loss failed without a negative energy balance. Many trends like gene-based or microbiome-based dietary recommendations still lack conclusive scientific evidence. In general, weight loss studies often have methodological limitations (e.g., study design or duration), leading to results not being comparable, and they therefore should be interpreted with caution. With lifestyle changes, a moderate weight loss after one year is possible. Other approaches, such as bariatric surgery, lead to greater weight loss, but are proven only for specific target groups. More research, especially by long-term intervention studies, is needed to evaluate weight loss concepts and to obtain evidence-based tailored recommendations.

## Figures and Tables

**Table 1:** Body mass index and waist circumference: cut-offs and risk of co-morbidities.

Body Mass Index			
	18.5–24.9 kg/m^2^	25–29.9 kg/m^2^	≥30 kg/m^2^
Classification	Normal weight	Overweight	Obese
Risk of co-morbidities	Low	Increased	High
**Waist circumference**			
	Men: <94 cm	Men: 94–101.9 cm	Men: ≥102 cm
	Women: <80 cm	Women: 80–87.9 cm	Women: ≥88 cm
Classification	Normal fat distribution	Moderate central fat accumulation	High central fat accumulation
Risk of co-morbidities	Low	Increased	High

**Table 2:** Practical recommendations and examples to reduce energy intake.

Item	Recommendation	Implementation
Energy density	**Prefer low energy dense food.** Choose food with lower energy content per 100 g.	Salami → ham Rice → potatoes Cream cake → fruit tart
Dietary fat	**Watch out for hidden fats.** Choose food low in fat, or pick the fat-reduced alternative. **Prefer fat-reduced food preparing methods.** Choose methods without adding fat.	Potato chips → pretzel sticks Butter, cream cheese → low fat-cream cheese Frying → braising Roasting → cooking Chips → boiled potatoes
Plant-based food	**Take five portions a day** **of vegetables and fruits.** Choose plant-based food instead of meat.	Wheat noodles → zucchini pasta Mascarpone cream → fresh fruit for dessert Meat bolognese → vegetable bolognese
Dietary fiber	**Increase dietary fibre intake to 25 to 30 g a day.** Choose whole grain products, legumes, vegetables, fruits.	White noodles → whole grain noodles White bread → whole grain bread Rice → legumes
Beverages	**Drink caloric-free beverages.** Choose beverages without sugar and calories.	Water and tea Coffee without sugar Beverages with artificial sweeteners instead of sugar-sweetened beverages

## References

[1] World Health Organization (2000). Obesity: Preventing and Managing the Global Epidemic: Report of a WHO Consultation. WHO Technical Report Series 894.

[2] Waxman A. (2005). Why a global strategy on diet, physical activity and health?. World Rev. Nutr. Diet..

[3] World Health Organization Obesity and Overweight. https://www.who.int/en/news-room/fact-sheets/detail/obesity-and-overweight.

[4] Guh D.P., Zhang W., Bansback N., Amarsi Z., Birmingham C.L., Anis A.H. (2009). The incidence of co-morbidities related to obesity and overweight: A systematic review and meta-analysis. BMC Public Health.

[5] Gao M., Piernas C., Astbury N.M., Hippisley-Cox J., O’Rahilly S., Aveyard P., Jebb S.A. (2021). Associations between body-mass index and COVID-19 severity in 6·9 million people in England: A prospective, community-based, cohort study. Lancet Diabetes Endocrinol..

[6] Blüher M. (2019). Obesity: Global epidemiology and pathogenesis. Nat. Rev. Endocrinol..

[7] Fontaine K.R., Redden D.T., Wang C., Westfall A.O., Allison D.B. (2003). Years of life lost due to obesity. JAMA.

[8] Whitlock G., Lewington S., Sherliker P., Clarke R., Emberson J., Halsey J., Qizilbash N., Collins R., Peto R. (2009). Body-mass index and cause-specific mortality in 900 000 adults: Collaborative analyses of 57 prospective studies. Lancet.

[9] Yumuk V., Tsigos C., Fried M., Schindler K., Busetto L., Micic D., Toplak H. (2015). European Guidelines for Obesity Management in Adults. Obes. Facts.

[10] Hill J.O., Wyatt H.R., Peters J.C. (2012). Energy balance and obesity. Circulation.

[11] Camacho S., Ruppel A. (2017). Is the calorie concept a real solution to the obesity epidemic?. Glob. Health Action.

[12] Wright J.D., Kennedy-Stephenson J., Wang C.Y., McDowell M.A., Johnson C.L. (2004). Trends in Intake of Energy and Macronutrients—United States, 1971–2000. JAMA.

[13] Bassett D.R., Wyatt H.R., Thompson H., Peters J.C., Hill J.O. (2010). Pedometer-measured physical activity and health behaviors in U.S. adults. Med. Sci. Sports Exerc..

[14] Church T.S., Thomas D.M., Tudor-Locke C., Katzmarzyk P.T., Earnest C.P., Rodarte R.Q., Martin C.K., Blair S.N., Bouchard C. (2011). Trends over 5 decades in U.S. occupation-related physical activity and their associations with obesity. PLoS ONE.

[15] Rolls B.J. (2003). The Supersizing of America: Portion Size and the Obesity Epidemic. Nutr. Today.

[16] Yanovski J.A. (2018). Obesity: Trends in underweight and obesity—Scale of the problem. Nat. Rev. Endocrinol..

[17] Abarca-Gómez L., Abdeen Z.A., Hamid Z.A., Abu-Rmeileh N.M., Acosta-Cazares B., Acuin C., Adams R.J., Aekplakorn W., Afsana K., Aguilar-Salinas C.A. (2017). Worldwide trends in body-mass index, underweight, overweight, and obesity from 1975 to 2016: A pooled analysis of 2416 population-based measurement studies in 128·9 million children, adolescents, and adults. Lancet.

[18] NCD Risk Factor Collaboration (2016). Trends in adult body-mass index in 200 countries from 1975 to 2014: A pooled analysis of 1698 population-based measurement studies with 19·2 million participants. Lancet.

[19] Afshin A., Forouzanfar M.H., Reitsma M.B., Sur P., Estep K., Lee A., Marczak L., Mokdad A.H., Moradi-Lakeh M., Naghavi M. (2017). Health Effects of Overweight and Obesity in 195 Countries over 25 Years. N. Engl. J. Med..

[20] Smith K.B., Smith M.S. (2016). Obesity Statistics. Prim. Care.

[21] Dicken S.J., Mitchell J.J., Le Newberry Vay J., Beard E., Kale D., Herbec A., Shahab L. (2021). Impact of COVID-19 Pandemic on Weight and BMI among UK Adults: A Longitudinal Analysis of Data from the HEBECO Study. Nutrients.

[22] Seal A., Schaffner A., Phelan S., Brunner-Gaydos H., Tseng M., Keadle S., Alber J., Kiteck I., Hagobian T. (2021). COVID-19 pandemic and stay-at-home mandates promote weight gain in US adults. Obesity.

[23] Stefan N., Birkenfeld A.L., Schulze M.B. (2021). Global pandemics interconnected—Obesity, impaired metabolic health and COVID-19. Nat. Rev. Endocrinol..

[24] Lemstra M., Bird Y., Nwankwo C., Rogers M., Moraros J. (2016). Weight loss intervention adherence and factors promoting adherence: A meta-analysis. Patient Prefer. Adherence.

[25] European Food Safety Authority EFSA Sets European Dietary Reference Values for Nutrient Intakes. https://www.efsa.europa.eu/en/press/news/nda100326.

[26] European Food Safety Authority (2012). Scientific Opinion on Dietary Reference Values for protein. EFSA J..

[27] Shai I., Schwarzfuchs D., Henkin Y., Shahar D.R., Witkow S., Greenberg I., Golan R., Fraser D., Bolotin A., Vardi H. (2008). Weight loss with a low-carbohydrate, Mediterranean, or low-fat diet. N. Engl. J. Med..

[28] Sacks F.M., Bray G.A., Carey V.J., Smith S.R., Ryan D.H., Anton S.D., McManus K., Champagne C.M., Bishop L.M., Laranjo N. (2009). Comparison of weight-loss diets with different compositions of fat, protein, and carbohydrates. N. Engl. J. Med..

[29] Seidelmann S.B., Claggett B., Cheng S., Henglin M., Shah A., Steffen L.M., Folsom A.R., Rimm E.B., Willett W.C., Solomon S.D. (2018). Dietary carbohydrate intake and mortality: A prospective cohort study and meta-analysis. Lancet Public Health.

[30] Gjuladin-Hellon T., Davies I.G., Penson P., Amiri Baghbadorani R. (2019). Effects of carbohydrate-restricted diets on low-density lipoprotein cholesterol levels in overweight and obese adults: A systematic review and meta-analysis. Nutr. Rev..

[31] Austin G.L., Ogden L.G., Hill J.O. (2011). Trends in carbohydrate, fat, and protein intakes and association with energy intake in normal-weight, overweight, and obese individuals: 1971-2006. Am. J. Clin. Nutr..

[32] Manheimer E.W., van Zuuren E.J., Fedorowicz Z., Pijl H. (2015). Paleolithic nutrition for metabolic syndrome: Systematic review and meta-analysis. Am. J. Clin. Nutr..

[33] Mellberg C., Sandberg S., Ryberg M., Eriksson M., Brage S., Larsson C., Olsson T., Lindahl B. (2014). Long-term effects of a Palaeolithic-type diet in obese postmenopausal women: A 2-year randomized trial. Eur. J. Clin. Nutr..

[34] Johnston B.C., Kanters S., Bandayrel K., Wu P., Naji F., Siemieniuk R.A., Ball G.D.C., Busse J.W., Thorlund K., Guyatt G. (2014). Comparison of weight loss among named diet programs in overweight and obese adults: A meta-analysis. JAMA.

[35] Gardner C.D., Trepanowski J.F., Del Gobbo L.C., Hauser M.E., Rigdon J., Ioannidis J.P.A., Desai M., King A.C. (2018). Effect of Low-Fat vs Low-Carbohydrate Diet on 12-Month Weight Loss in Overweight Adults and the Association With Genotype Pattern or Insulin Secretion: The DIETFITS Randomized Clinical Trial. JAMA.

[36] Churuangsuk C., Kherouf M., Combet E., Lean M. (2018). Low-carbohydrate diets for overweight and obesity: A systematic review of the systematic reviews. Obes. Rev..

[37] Jenkins D.J.A., Wong J.M.W., Kendall C.W.C., Esfahani A., Ng V.W.Y., Leong T.C.K., Faulkner D.A., Vidgen E., Greaves K.A., Paul G. (2009). The effect of a plant-based low-carbohydrate (“Eco-Atkins”) diet on body weight and blood lipid concentrations in hyperlipidemic subjects. Arch. Intern. Med..

[38] Jenkins D.J.A., Wong J.M.W., Kendall C.W.C., Esfahani A., Ng V.W.Y., Leong T.C.K., Faulkner D.A., Vidgen E., Paul G., Mukherjea R. (2014). Effect of a 6-month vegan low-carbohydrate (‘Eco-Atkins’) diet on cardiovascular risk factors and body weight in hyperlipidaemic adults: A randomised controlled trial. BMJ Open.

[39] Esposito K., Kastorini C.-M., Panagiotakos D.B., Giugliano D. (2011). Mediterranean diet and weight loss: Meta-analysis of randomized controlled trials. Metab. Syndr. Relat. Disord..

[40] Leslie W.S., Taylor R., Harris L., Lean M.E.J. (2016). Weight losses with low-energy formula diets in obese patients with and without type 2 diabetes: Systematic review and meta-analysis. Int. J. Obes..

[41] Astbury N.M., Piernas C., Hartmann-Boyce J., Lapworth S., Aveyard P., Jebb S.A. (2019). A systematic review and meta-analysis of the effectiveness of meal replacements for weight loss. Obes. Rev..

[42] Lean M.E.J., Leslie W.S., Barnes A.C., Brosnahan N., Thom G., McCombie L., Peters C., Zhyzhneuskaya S., Al-Mrabeh A., Hollingsworth K.G. (2018). Primary care-led weight management for remission of type 2 diabetes (DiRECT): An open-label, cluster-randomised trial. Lancet.

[43] Lean M.E.J., Leslie W.S., Barnes A.C., Brosnahan N., Thom G., McCombie L., Peters C., Zhyzhneuskaya S., Al-Mrabeh A., Hollingsworth K.G. (2019). Durability of a primary care-led weight-management intervention for remission of type 2 diabetes: 2-year results of the DiRECT open-label, cluster-randomised trial. Lancet Diabetes Endocrinol..

[44] Conley M., Le Fevre L., Haywood C., Proietto J. (2018). Is two days of intermittent energy restriction per week a feasible weight loss approach in obese males? A randomised pilot study. Nutr. Diet..

[45] Trepanowski J.F., Kroeger C.M., Barnosky A., Klempel M.C., Bhutani S., Hoddy K.K., Gabel K., Freels S., Rigdon J., Rood J. (2017). Effect of Alternate-Day Fasting on Weight Loss, Weight Maintenance, and Cardioprotection Among Metabolically Healthy Obese Adults: A Randomized Clinical Trial. JAMA Intern. Med..

[46] Cioffi I., Evangelista A., Ponzo V., Ciccone G., Soldati L., Santarpia L., Contaldo F., Pasanisi F., Ghigo E., Bo S. (2018). Intermittent versus continuous energy restriction on weight loss and cardiometabolic outcomes: A systematic review and meta-analysis of randomized controlled trials. J. Transl. Med..

[47] Harvie M.N., Pegington M., Mattson M.P., Frystyk J., Dillon B., Evans G., Cuzick J., Jebb S.A., Martin B., Cutler R.G. (2011). The effects of intermittent or continuous energy restriction on weight loss and metabolic disease risk markers: A randomized trial in young overweight women. Int. J. Obes..

[48] Sundfør T.M., Svendsen M., Tonstad S. (2018). Effect of intermittent versus continuous energy restriction on weight loss, maintenance and cardiometabolic risk: A randomized 1-year trial. Nutr. Metab. Cardiovasc. Dis..

[49] Harris L., Hamilton S., Azevedo L.B., Olajide J., de Brún C., Waller G., Whittaker V., Sharp T., Lean M., Hankey C. (2018). Intermittent fasting interventions for treatment of overweight and obesity in adults: A systematic review and meta-analysis. JBI Database System. Rev. Implement. Rep..

[50] Allaf M., Elghazaly H., Mohamed O.G., Fareen M.F.K., Zaman S., Salmasi A.-M., Tsilidis K., Dehghan A. (2021). Intermittent fasting for the prevention of cardiovascular disease. Cochrane Database Syst. Rev..

[51] Templeman I., Smith H.A., Chowdhury E., Chen Y.-C., Carroll H., Johnson-Bonson D., Hengist A., Smith R., Creighton J., Clayton D. (2021). A randomized controlled trial to isolate the effects of fasting and energy restriction on weight loss and metabolic health in lean adults. Sci. Transl. Med..

[52] Lowe D.A., Wu N., Rohdin-Bibby L., Moore A.H., Kelly N., Liu Y.E., Philip E., Vittinghoff E., Heymsfield S.B., Olgin J.E. (2020). Effects of Time-Restricted Eating on Weight Loss and Other Metabolic Parameters in Women and Men With Overweight and Obesity: The TREAT Randomized Clinical Trial. JAMA Intern. Med..

[53] Jamshed H., Beyl R.A., Della Manna D.L., Yang E.S., Ravussin E., Peterson C.M. (2019). Early Time-Restricted Feeding Improves 24-h Glucose Levels and Affects Markers of the Circadian Clock, Aging, and Autophagy in Humans. Nutrients.

[54] Sutton E.F., Beyl R., Early K.S., Cefalu W.T., Ravussin E., Peterson C.M. (2018). Early Time-Restricted Feeding Improves Insulin Sensitivity, Blood Pressure, and Oxidative Stress Even without Weight Loss in Men with Prediabetes. Cell Metab..

[55] Nas A., Mirza N., Hägele F., Kahlhöfer J., Keller J., Rising R., Kufer T.A., Bosy-Westphal A. (2017). Impact of breakfast skipping compared with dinner skipping on regulation of energy balance and metabolic risk. Am. J. Clin. Nutr..

[56] Mekary R.A., Giovannucci E., Willett W.C., van Dam R.M., Hu F.B. (2012). Eating patterns and type 2 diabetes risk in men: Breakfast omission, eating frequency, and snacking. Am. J. Clin. Nutr..

[57] Zeevi D., Korem T., Zmora N., Israeli D., Rothschild D., Weinberger A., Ben-Yacov O., Lador D., Avnit-Sagi T., Lotan-Pompan M. (2015). Personalized Nutrition by Prediction of Glycemic Responses. Cell.

[58] Loos R.J. (2018). The genetics of adiposity. Curr. Opin. Genet. Dev..

[59] Holzapfel C., Dawczynski C., Henze A., Simon M.C. (2021). Personalized dietary recommendations for weight loss. A scientific perspective from various angles. Ernahr. Umsch..

[60] Claussnitzer M., Dankel S.N., Kim K.-H., Quon G., Meuleman W., Haugen C., Glunk V., Sousa I.S., Beaudry J.L., Puviindran V. (2015). FTO Obesity Variant Circuitry and Adipocyte Browning in Humans. N. Engl. J. Med..

[61] Holzapfel C., Sag S., Graf-Schindler J., Fischer M., Drabsch T., Illig T., Grallert H., Stecher L., Strack C., Caterson I.D. (2021). Association between Single Nucleotide Polymorphisms and Weight Reduction in Behavioural Interventions-A Pooled Analysis. Nutrients.

[62] Celis-Morales C., Marsaux C.F., Livingstone K.M., Navas-Carretero S., San-Cristobal R., Fallaize R., Macready A.L., O’Donovan C., Woolhead C., Forster H. (2017). Can genetic-based advice help you lose weight? Findings from the Food4Me European randomized controlled trial. Am. J. Clin. Nutr..

[63] Ellis A., Rozga M., Braakhuis A., Monnard C.R., Robinson K., Sinley R., Wanner A., Vargas A.J. (2021). Effect of Incorporating Genetic Testing Results into Nutrition Counseling and Care on Health Outcomes: An Evidence Analysis Center Systematic Review-Part II. J. Acad. Nutr. Diet..

[64] Camp K.M., Trujillo E. (2014). Position of the Academy of Nutrition and Dietetics: Nutritional genomics. J. Acad. Nutr. Diet..

[65] Drabsch T., Holzapfel C. (2019). A Scientific Perspective of Personalised Gene-Based Dietary Recommendations for Weight Management. Nutrients.

[66] Bayer S., Winkler V., Hauner H., Holzapfel C. (2020). Associations between Genotype-Diet Interactions and Weight Loss-A Systematic Review. Nutrients.

[67] Khera A.V., Chaffin M., Aragam K.G., Haas M.E., Roselli C., Choi S.H., Natarajan P., Lander E.S., Lubitz S.A., Ellinor P.T. (2018). Genome-wide polygenic scores for common diseases identify individuals with risk equivalent to monogenic mutations. Nat. Genet..

[68] Khera A.V., Chaffin M., Wade K.H., Zahid S., Brancale J., Xia R., Distefano M., Senol-Cosar O., Haas M.E., Bick A. (2019). Polygenic Prediction of Weight and Obesity Trajectories from Birth to Adulthood. Cell.

[69] Kolodziejczyk A.A., Zheng D., Elinav E. (2019). Diet-microbiota interactions and personalized nutrition. Nat. Rev. Microbiol..

[70] Magni P., Bier D.M., Pecorelli S., Agostoni C., Astrup A., Brighenti F., Cook R., Folco E., Fontana L., Gibson R.A. (2017). Perspective: Improving Nutritional Guidelines for Sustainable Health Policies: Current Status and Perspectives. Adv. Nutr..

[71] von Schwartzenberg R.J., Bisanz J.E., Lyalina S., Spanogiannopoulos P., Ang Q.Y., Cai J., Dickmann S., Friedrich M., Liu S.-Y., Collins S.L. (2021). Caloric restriction disrupts the microbiota and colonization resistance. Nature.

[72] Breuninger T.A., Wawro N., Breuninger J., Reitmeier S., Clavel T., Six-Merker J., Pestoni G., Rohrmann S., Rathmann W., Peters A. (2021). Associations between habitual diet, metabolic disease, and the gut microbiota using latent Dirichlet allocation. Microbiome.

[73] Cani P.D., van Hul M., Lefort C., Depommier C., Rastelli M., Everard A. (2019). Microbial regulation of organismal energy homeostasis. Nat. Metab..

[74] DGAC Scientific Report of the 2015 Dietary Guidelines Advisory Committee. https://health.gov/sites/default/files/2019-09/Scientific-Report-of-the-2015-Dietary-Guidelines-Advisory-Committee.pdf.

[75] Jebb S.A., Ahern A.L., Olson A.D., Aston L.M., Holzapfel C., Stoll J., Amann-Gassner U., Simpson A.E., Fuller N.R., Pearson S. (2011). Primary care referral to a commercial provider for weight loss treatment versus standard care: A randomised controlled trial. Lancet.

[76] Holzapfel C., Cresswell L., Ahern A.L., Fuller N.R., Eberhard M., Stoll J., Mander A.P., Jebb S.A., Caterson I.D., Hauner H. (2014). The challenge of a 2-year follow-up after intervention for weight loss in primary care. Int. J. Obes..

[77] Johnston C.A., Rost S., Miller-Kovach K., Moreno J.P., Foreyt J.P. (2013). A randomized controlled trial of a community-based behavioral counseling program. Am. J. Med..

[78] Ard J.D., Lewis K.H., Rothberg A., Auriemma A., Coburn S.L., Cohen S.S., Loper J., Matarese L., Pories W.J., Periman S. (2019). Effectiveness of a Total Meal Replacement Program (OPTIFAST Program) on Weight Loss: Results from the OPTIWIN Study. Obesity.

[79] Harvey J., Krukowski R., Priest J., West D. (2019). Log Often, Lose More: Electronic Dietary Self-Monitoring for Weight Loss. Obesity.

[80] Jensen M.D., Ryan D.H., Apovian C.M., Ard J.D., Comuzzie A.G., Donato K.A., Hu F.B., van Hubbard S., Jakicic J.M., Kushner R.F. (2014). 2013 AHA/ACC/TOS Guideline for the Management of Overweight and Obesity in Adults. Circulation.

[81] Patel M.L., Wakayama L.N., Bennett G.G. (2021). Self-Monitoring via Digital Health in Weight Loss Interventions: A Systematic Review Among Adults with Overweight or Obesity. Obesity.

[82] Michie S., Abraham C., Whittington C., McAteer J., Gupta S. (2009). Effective techniques in healthy eating and physical activity interventions: A meta-regression. Health Psychol..

[83] Harkin B., Webb T.L., Chang B.P.I., Prestwich A., Conner M., Kellar I., Benn Y., Sheeran P. (2016). Does monitoring goal progress promote goal attainment? A meta-analysis of the experimental evidence. Psychol. Bull..

[84] Spring B., Champion K.E., Acabchuk R., Hennessy E.A. (2021). Self-regulatory behaviour change techniques in interventions to promote healthy eating, physical activity, or weight loss: A meta-review. Health Psychol. Rev..

[85] Carter M.C., Burley V.J., Nykjaer C., Cade J.E. (2013). Adherence to a smartphone application for weight loss compared to website and paper diary: Pilot randomized controlled trial. J. Med. Internet Res..

[86] Stubbs R.J., Duarte C., Palmeira A.L., Sniehotta F.F., Horgan G., Larsen S.C., Marques M.M., Evans E.H., Ermes M., Harjumaa M. (2021). Evidence-Based Digital Tools for Weight Loss Maintenance: The NoHoW Project. Obes. Facts.

[87] Turner-McGrievy G., Tate D. (2011). Tweets, Apps, and Pods: Results of the 6-month Mobile Pounds Off Digitally (Mobile POD) randomized weight-loss intervention among adults. J. Med. Internet Res..

[88] Wharton C.M., Johnston C.S., Cunningham B.K., Sterner D. (2014). Dietary self-monitoring, but not dietary quality, improves with use of smartphone app technology in an 8-week weight loss trial. J. Nutr. Educ. Behav..

[89] Villinger K., Wahl D.R., Boeing H., Schupp H.T., Renner B. (2019). The effectiveness of app-based mobile interventions on nutrition behaviours and nutrition-related health outcomes: A systematic review and meta-analysis. Obes. Rev..

[90] Roess A. (2017). The Promise, Growth, and Reality of Mobile Health—Another Data-free Zone. N. Engl. J. Med..

[91] Svetkey L.P., Batch B.C., Lin P.-H., Intille S.S., Corsino L., Tyson C.C., Bosworth H.B., Grambow S.C., Voils C., Loria C. (2015). Cell phone intervention for you (CITY): A randomized, controlled trial of behavioral weight loss intervention for young adults using mobile technology. Obesity.

[92] Bardus M., van Beurden S.B., Smith J.R., Abraham C. (2016). A review and content analysis of engagement, functionality, aesthetics, information quality, and change techniques in the most popular commercial apps for weight management. Int. J. Behav. Nutr. Phys. Act..

[93] Rivera J., McPherson A., Hamilton J., Birken C., Coons M., Iyer S., Agarwal A., Lalloo C., Stinson J. (2016). Mobile Apps for Weight Management: A Scoping Review. JMIR Mhealth Uhealth.

[94] Pilitsi E., Farr O.M., Polyzos S.A., Perakakis N., Nolen-Doerr E., Papathanasiou A.-E., Mantzoros C.S. (2019). Pharmacotherapy of obesity: Available medications and drugs under investigation. Metabolism.

[95] Daneschvar H.L., Aronson M.D., Smetana G.W. (2016). FDA-Approved Anti-Obesity Drugs in the United States. Am. J. Med..

[96] Tak Y.J., Lee S.Y. (2021). Anti-Obesity Drugs: Long-Term Efficacy and Safety: An Updated Review. World J. Mens. Health.

[97] Apovian C.M., Aronne L.J., Bessesen D.H., McDonnell M.E., Murad M.H., Pagotto U., Ryan D.H., Still C.D. (2015). Pharmacological management of obesity: An endocrine Society clinical practice guideline. J. Clin. Endocrinol. Metab..

[98] May M., Schindler C., Engeli S. (2020). Modern pharmacological treatment of obese patients. Ther. Adv. Endocrinol. Metab..

[99] Khera R., Murad M.H., Chandar A.K., Dulai P.S., Wang Z., Prokop L.J., Loomba R., Camilleri M., Singh S. (2016). Association of Pharmacological Treatments for Obesity With Weight Loss and Adverse Events: A Systematic Review and Meta-analysis. JAMA.

[100] Welbourn R., Hollyman M., Kinsman R., Dixon J., Liem R., Ottosson J., Ramos A., Våge V., Al-Sabah S., Brown W. (2019). Bariatric Surgery Worldwide: Baseline Demographic Description and One-Year Outcomes from the Fourth IFSO Global Registry Report 2018. Obes. Surg..

[101] Puzziferri N., Roshek T.B., Mayo H.G., Gallagher R., Belle S.H., Livingston E.H. (2014). Long-term follow-up after bariatric surgery: A systematic review. JAMA.

[102] O’Brien P.E., Hindle A., Brennan L., Skinner S., Burton P., Smith A., Crosthwaite G., Brown W. (2019). Long-Term Outcomes After Bariatric Surgery: A Systematic Review and Meta-analysis of Weight Loss at 10 or More Years for All Bariatric Procedures and a Single-Centre Review of 20-Year Outcomes After Adjustable Gastric Banding. Obes. Surg..

[103] Toussi R., Fujioka K., Coleman K.J. (2009). Pre- and postsurgery behavioral compliance, patient health, and postbariatric surgical weight loss. Obesity.

[104] Benalcazar D.A., Cascella M. (2021). StatPearls: Obesity Surgery Pre-Op Assessment And Preparation.

[105] Lupoli R., Lembo E., Saldalamacchia G., Avola C.K., Angrisani L., Capaldo B. (2017). Bariatric surgery and long-term nutritional issues. World J. Diabetes.

[106] Di Lorenzo N., Antoniou S.A., Batterham R.L., Busetto L., Godoroja D., Iossa A., Carrano F.M., Agresta F., Alarçon I., Azran C. (2020). Clinical practice guidelines of the European Association for Endoscopic Surgery (EAES) on bariatric surgery: Update 2020 endorsed by IFSO-EC, EASO and ESPCOP. Surg. Endosc..

